# Promoting stress and anxiety recovery in older adults: assessing the therapeutic influence of biophilic green walls and outdoor view

**DOI:** 10.3389/fpubh.2024.1352611

**Published:** 2024-04-15

**Authors:** Su Xiaoxue, Xuan Huang

**Affiliations:** School of Architecture, Southwest Jiaotong University, Chengdu, China

**Keywords:** nature connectedness, older adults, indoor public activity spaces, virtual reality (VR), physiological responses, biometric monitoring sensors

## Abstract

Previous research has already provided evidence regarding the favorable impact of green walls and outdoor views on stress reduction and anxiety alleviation. However, there has been limited exploration into the combined effects of green walls and outdoor views on older adults. In this study, a between-subjects experiment was conducted using virtual reality (VR) technology with 23 participants. Following exposure to stressors, each participant underwent four randomized sessions, each lasting 5 min, in various virtual reality (VR) environments, encompassing non-biophilic and biophilic environments (including green walls, outdoor views, and their combination). Throughout the process, we measured physiological indicators of stress responses, including heart rate, heart rate variability, skin conductance levels, and blood pressure, using biometric sensors. Psychological changes in participants, including anxiety levels, were evaluated through the State–Trait Anxiety Inventory, recovery scales, and self-reported emotional assessments. In conclusion, in comparison to non-biophilic environments, older adults consistently exhibited lower stress levels, experienced superior anxiety relief, and demonstrated improved recovery in nature connectedness environments, with a notably faster recovery rate. These findings suggest that the incorporation of nature connectedness principles into the indoor environments of public activity spaces within older adults care facilities can significantly contribute to stress reduction and anxiety alleviation among older adults. Furthermore, these effects appear to be contingent on the specific types of nature connectedness environments. These results can provide substantial evidence to support the design of indoor common activity spaces within older adults care facilities.

## 1 Introduction

The global population is currently experiencing a rapid aging trend (1), with the growing older adults demographic expected to have significant impacts on both society and healthcare systems (2). In light of this demographic transition, there is a growing emphasis on addressing the mental health that older adults face (3). Issues such as social isolation, loss of independence, loneliness, and psychological distress can lead to stress and anxiety, ultimately affecting the mental and physical well-being of older adults individuals. Surveys have shown that approximately 3.8% of the older adults population is affected by anxiety disorders. Furthermore, the significance of older adults care facilities is increasingly acknowledged. The environmental factors within these facilities play a pivotal role in shaping the quality of life and health of older adults (4). However, several obstacles exist within the environment of older adults care facilities, including limited opportunities for older adults to connect with the natural world (5). Additionally, the prevalence of diseases related to psychological stress is on the rise among older adults (6, 7). Therefore, investigating how to integrate nature into indoor public spaces in older adults care facilities, and enhance the psychological well-being and overall quality of life for older adults, is of paramount importance.

Nature Connectedness is a concept rooted in the biophilia theory. In 1964, Erich Fromm (8) first introduced the term “biophilia” in his book “The Heart of Man: Its Genius for Good and Evil.” Subsequently, in 1984, Edward O. Wilson (9) proposed the “Biophilia Hypothesis” in his book “Biophilia,” suggesting that humans have an innate emotional connection and affinity with nature. In 1982, Roger Ulrich introduced the “Stress Reduction Theory,” which underscores the role of natural elements in activating the parasympathetic nervous system. This activation leads to reductions in heart rate, blood pressure, skin conductance, and salivary cortisol levels, ultimately alleviating psychological stress and promoting physiological relaxation (10). Additionally, S. Kaplan and R. Kaplan presented the “Attention Restoration Theory” (11), emphasizing that natural environments, by providing captivating stimuli, assist individuals in regaining directed attention, thereby facilitating psychological restoration experiences (12, 13). Numerous studies have robustly demonstrated that nature connectedness has a positive impact on mental and physical health (14–16). Nature has a beneficial impact on human health (17–19). Extensive research indicates that being close to nature and experiencing positive interactions in natural environments can trigger beneficial psychological and physiological responses (13, 20–22). These studies consistently emphasize a positive correlation between improved psychological and physiological responses and the connection to nature (23). For instance, the outdoor views and green walls positively influence behavior and emotional states through visual stimuli (24). Moreover, research has consistently identified a significant association between outdoor view and mental health (25–28). Green walls, with their potential for health benefits (29, 30), enhance the environment and positively affecting emotions and well-being (31, 32). While some research has explored the health benefits of green walls and outdoor views, there is relatively limited research on the combined effects of green walls and outdoor views. Quantitative studies examining the physiological and psychological responses of older adults (33) to green walls, outdoor views, and their combination are scarce. Investigating the design of indoor public activity spaces in older adults care facilities holds significant importance.

These initial investigations have their limitations. Firstly, environmental exposure studies typically focus on individual nature elements’ impact, such as the impact of green walls or outdoor views on individuals (34, 35), and the combined effects of these elements have yet to be studied. Secondly, while there have been studies quantifying short-term physiological changes in response to environmental exposure using VR and biometric monitoring sensors (36, 37), research on the older adults population in this context is relatively scarce (38). Lastly, there is a notable scarcity of research examining the stress recovery and anxiety alleviation in older adults related to different biophilic elements (green walls, outdoor views, and combination).

To further investigate the correlation between Nature Connectedness and older adults’ responses, this investigation employs encompassing virtual reality (VR) and wearable biometric monitoring sensors, in conjunction with rigorously validated psychological assessments, deployed for the quantification of participants’ physiological and psychological reactions within diverse nature connectedness public activity spaces (green walls, outdoor views, and combinations). Our principal objective is to ascertain whether, in contrast to non-biophilic environments, green walls, outdoor views, or their amalgamation demonstrate heightened efficacy in mitigating stress and anxiety among older adults. Concurrently, we explore the diverse influences exerted by various categories of biophilic environments on physiological and psychological responses. The study endeavors to elucidate the latent favorable impacts of green walls and outdoor views on the psychological well-being of older adults, while also scrutinizing the timeliness of virtual reality technology in evaluating these effects.

## 2 Methods

### 2.1 Participants

In the months of June to August 2023, we successfully recruited a cohort of 25 senior individuals (aged ≥60) to partake in our research initiative. These participants willingly volunteered for the experiment, and each received a compensation of approximately 100 yuan. Rigorous pre-screening measures were implemented to exclude individuals undergoing stress treatments or those taking stress-controlling medications. It is important to note that all participants were fully informed and provided their consent for the utilization of physiological data.

### 2.2 Study design

A randomized crossover study design was used in this study. In light of the limited participant pool, a self-control approach was adopted to mitigate potential confounding variables and achieve statistical equivalence with a parallel design. Each participant engaged with the four distinct environments—non-biophilic, green walls, outdoor view, and combination of green walls and outdoor view—on separate dates. These engagements were randomized and scheduled on the same day and time within a week to mitigate potential influences of circadian rhythms on physiological and psychological responses (39).

### 2.3 Environmental exposure

**Figure 1:**
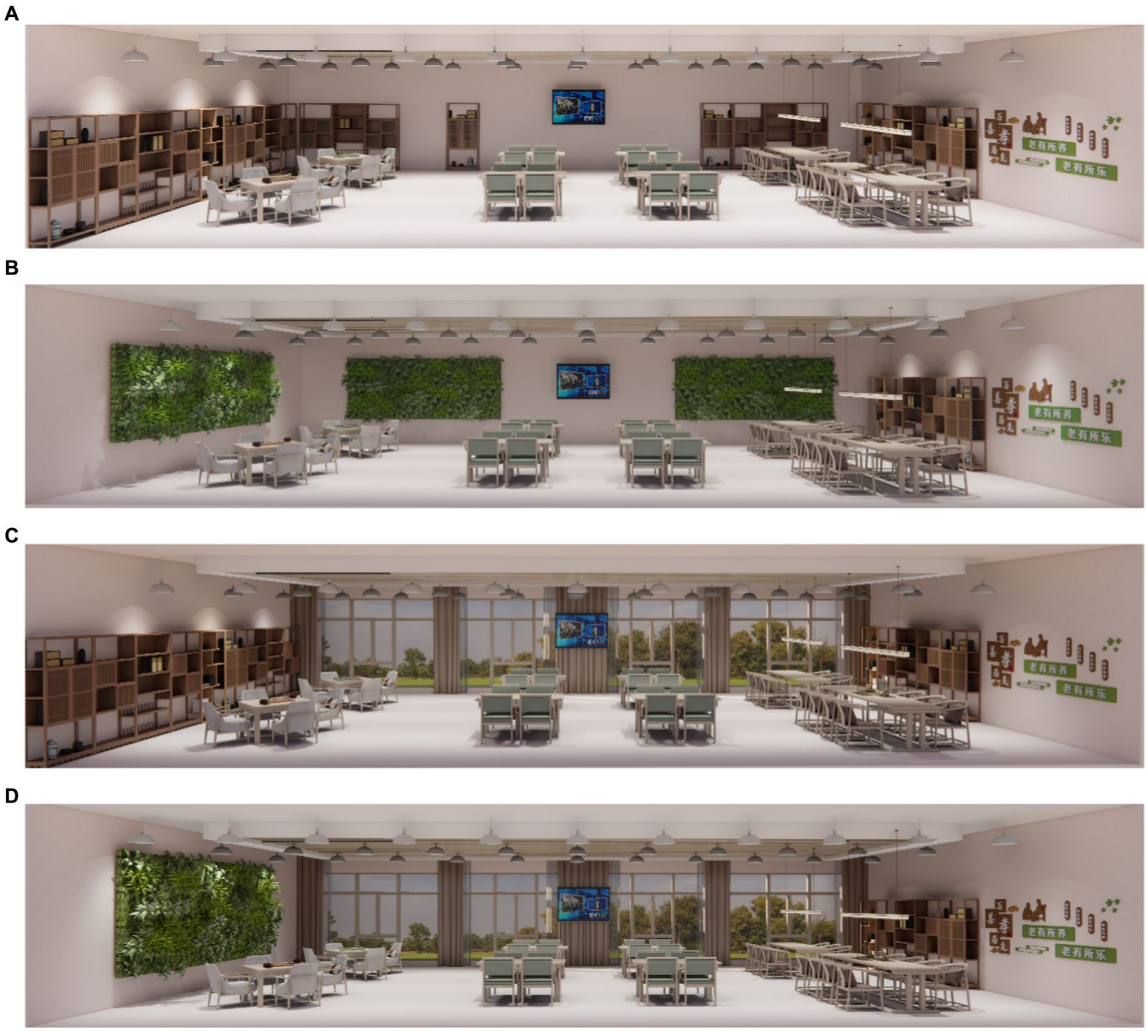
Four virtual reality office layouts. **(A)** Non-biophilic, **(B)** Green wall, **(C)** Outdoor view, **(D)** Combination.

To assess participants’ responses within different biophilic environments, a preliminary design was created in SketchUp, comprising one non-biophilic environment and three distinct biophilic environments (Figure 1). During the experiment, Enscape (version 3.4) was employed for real-time rendering, utilizing VR technology to simulate authentic scenarios. The experiment incorporated two biophilic elements: “green walls” and “outdoor view.” Firstly, the interior public environments of the older adults care facility were categorized into “windowed” and “windowless” variations. Secondly, research indicated that natural landscape interaction held primary importance in the preferences of seniors. Following this, variable green walls were ranked as a secondary preference. Specifically, the green walls were integrated within the indoor setting, while outdoor view were conveyed through panoramic views of trees, grasslands, and sunlight. Additionally, an environment combining both green walls and outdoor view was designed, with a non-biophilic environment serving as a control design. Throughout the experiment, all four environments were maintained at uniform dimensions and comparable layouts, maximizing their comparability. Apart from alterations in biophilic elements, the four environments remained consistent in all other aspects.

### 2.4 Outcome measures

#### 2.4.1 Physiological indicators of stress

Participants’ physiological indicators of acute stress were measured using biometric sensors, including heart rate (HR), heart rate variability (HRV), skin conductance level (SCL), and blood pressure (BP). In the stress recovery experiments, participants wore the Polar H10 - Verity Sense heart rate strap, which continuously collected raw electrocardiogram (ECG) data. HR and HRV were computed from this data. Specifically, HRV data were analyzed using Kubios HRV Standard software, which extracted time-domain and frequency-domain features from the RR interval data recorded in the HRV data. Kubios HRV Standard employed threshold-based beat-to-beat correction to remove artifacts and performed short-term HRV analysis at 30-s intervals, the minimum time resolution at which the sensor computed HRV. HR output was also averaged at 30-s intervals. HRV metrics included time-domain parameters (SDNN, RMSSD) and frequency-domain parameters (LF and HF power, peak frequency, and LF/HF ratio) (22). Time-domain analysis included SDNN, which represents the standard deviation of R-R intervals. A decrease in SDNN suggests reduced autonomic nervous system (ANS) regulation capacity, indicating a state of heightened anxiety and stress (40). Lower SDNN values are often associated with decreased ability for ANS to effectively regulate physiological processes (41), potentially leading to heightened sympathetic nervous system (SNS) activity (“fight or flight” response) and reduced parasympathetic nervous system (PNS) activity (“rest and digest” response), resulting in increased anxiety and tension (42). RMSSD, or root mean square of successive differences, is related to parasympathetic nervous system (PNS) activity and is a time-domain metric used to assess HRV (43). Higher RMSSD values typically indicate better PNS function and increased HRV (44), often associated with improved cardiovascular health and stress reduction (45, 46). Frequency-domain analysis included LF (low-frequency) and HF (high-frequency) power, with LF representing power in the low-frequency range (0.04–0.15 Hz) and HF representing power in the high-frequency range (0.15–0.4 Hz) (47). Skin conductance measurements (SCL in μs) were collected using the BIOPAC MP150 electrodermal activity (EDA) system and placed on the participants’ left fingers and wrists. SCL data were averaged every 30 s to match the output interval of the electrocardiogram sensor. Research has shown that individuals with higher baseline SCL levels may exhibit traits such as introversion, nervousness, anxiety, emotional instability, and heightened reactivity to stimuli (48, 49). Blood pressure measurements, including systolic blood pressure (SBP) and diastolic blood pressure [DBP(mmHg)] were obtained using the OMRON EVOLV all-in-one upper arm electronic blood pressure monitor. These measurements were taken after the baseline assessment, immediately after the stress induction task (pre-recovery), and after a 5-min recovery period (post-recovery).

#### 2.4.2 Psychological indicators of stress

In addition, psychological indicators of anxiety levels were measured using self-report instruments, including the State–Trait Anxiety Inventory (STAI), an environmental satisfaction and perceived restorativeness questionnaire, and a self-reported emotional assessment. The State–Trait Anxiety Inventory (STAI) was selected as the tool for assessing the impact of nature connectedness environments on psychological states. The STAI is a reliable, effective, and user-friendly anxiety measurement tool consisting of 20 items with four response options: “not at all,” “somewhat,” “moderately,” and “very much.” It is primarily used to reflect immediate or recent experiences of fear, tension, worry, and nervousness and can assess anxiety levels under stressful conditions. This experiment employed Likert-scale questionnaires to gather attitude feedback from participants and collect data on environmental satisfaction. To evaluate the restorative effects subjectively perceived in different environments, a simplified version of the Perceived restorativeness scale, consisting of six items on a 7-point scale, was administered. Participants were asked to provide self-assessments of their emotional changes, including stress, depression, and excitement, both before the experiment (baseline phase) and after exposure to the test environments. Self-reported emotional changes were collected for analysis.

### 2.5 Procedures

**Figure 2:**
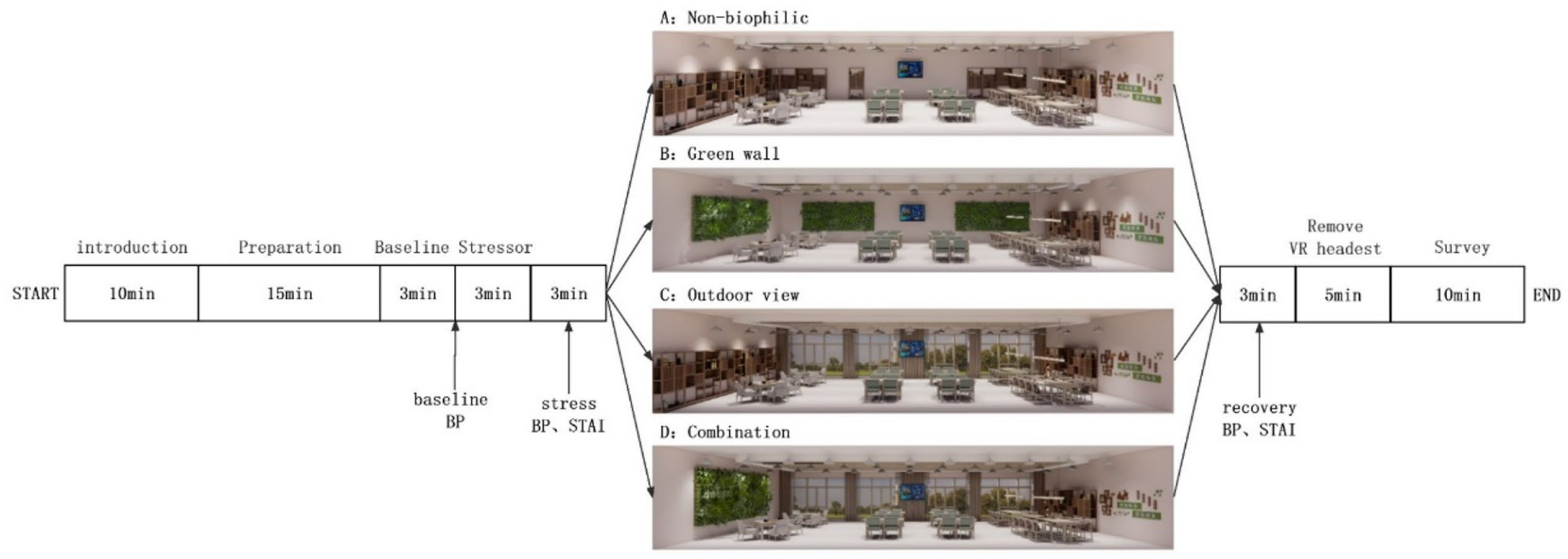
Experimental procedure. BP, Blood Pressure; HR, Heart Rate; STAI, State–Trait Anxiety Inventory; VR, Virtual Reality.

The experiment was divided into three stages (Figure 2): Preparation and Baseline, Stress, and Recovery. During the Preparation stage, participants were introduced to the experiment’s purpose, provided informed consent for the use of physiological and psychological data, and familiarized themselves with the virtual reality (VR) setup and bio-monitoring sensors. Participants were given 5 min to acquaint themselves with VR and understand the procedures for psychological testing. Subsequently, participants had a 10-min rest period, during which their baseline physiological measurements were recorded. In the Stress phase, participants were required to complete two stress-inducing tasks: the Digit Span Test and the Fancier Mackworth Test. In the Digit Span Test, a series of numbers appeared on the computer screen one after another, each displayed for only 1 s. After each series of numbers, participants had 20 s to select the numbers in sequence. Each participant completed one round of this task, with the number of digits increasing from two to nine. In the Fancier Mackworth Test, the clock hand would jump at random intervals, and participants needed to click on the circle within 1 s. Incorrect clicks or failure to click would result in a red warning. This task lasted for 3 min.

Following the completion of the stress-inducing tasks, participants underwent pre-recovery assessments, including the State–Trait Anxiety Inventory (STAI), self-reported emotional testing, and measurements of blood pressure and heart rate. Subsequently, participants were randomly assigned to one of four virtual public activity spaces, where they observed the layout of the virtual environment. Afterward, measurements of blood pressure and heart rate were taken again, and post-recovery assessments, including STAI, self-reported emotions, environmental satisfaction, and perceived restorativeness testing, were conducted. Finally, participants were relieved of the experimental equipment, and assistance was provided for them to complete a baseline survey. This survey included questions about their demographic information (age, gender), health status (excellent, very good, good, fair, poor), sleep quality (good/bad), consumption of caffeinated beverages (yes/no), smoking (yes/no), alcohol consumption (yes/no), high-intensity exercise (yes/no), and stress level (rated from 1 to 5, with 1 being low and 5 being high). Participants also ranked their preferences for “Non-Biophilic,” “Green Wall,” “Outdoor view,” and “Combination of Green Wall and Outdoor view,” as well as provided ratings on a scale of 0 to 10 based on the Likert scale. The baseline survey was conducted at the end of the experiment to avoid disclosing the experiment’s objectives. The entire experiment lasted approximately 60 min.

### 2.6 Statistical analyses

To ensure the effectiveness of the experiment, participants engaged in four different environments at the same time each week. To test the effectiveness of the random assignment, a one-way analysis of variance (ANOVA) was conducted to examine whether the baseline, physiological measurements, stress, and anxiety levels across the four conditions were similar, with statistical significance determined at *p* < 0.05/*p* > 0.05. To determine the effectiveness of the stress induction tasks, independent sample t-tests were employed for normally distributed data, and the Wilcoxon signed-rank test was used for non-normally distributed data. This was done to confirm whether participants’ stress levels were significantly higher than their baseline measurements following stress induction, with statistical significance set at *p* < 0.05/*p* > 0.05. Since the measurement data consisted of repeated measurements for each participant, participant IDs were used to identify repeated observations for the same individual.

Using the changes in blood pressure (BP) and State–Trait Anxiety Inventory (STAI) scores as dependent variables in a multivariate linear regression model, the non-biophilic environment was used as the reference (Model 1). During participation, baseline measurements may vary for the same participant. It was assumed that factors affecting changes in baseline measurements, including pre-experiment sleep, consumption of caffeinated beverages, smoking, alcohol consumption, high-intensity exercise, and pre-experiment stress levels, had the same impact on repeated measurements for the same participant during the experiment. Therefore, the regression model can be expressed as follows:

$\begin{array}{l} \Delta Yi=\beta 0+\left(\beta ₁\mathrm{Pressuriei}+\beta ₂\mathrm{Recoveryi}\right)\\ \;\mathrm{Environmenti}+ei \end{array}$ (1)

Where:

ΔYᵢ: The change in blood pressure for participant i, representing the difference in blood pressure between post-stress and recovery periods and blood pressure at 0 min.

Environmentᵢ: The impact of environmental factors on participant i’s changes in BP and STAI scores, with i indicating the environmental category in which the participant was placed (non-biophilic, green wall, outdoor view, combination of green wall and outdoor view).

β₁Pressureᵢ: The effect of stress on participant i’s changes in BP and STAI scores.

β₂Recoveryᵢ: The effect of the recovery period on participant i’s BP and STAI score changes.

eᵢ: Represents the error term associated with participant i, indicating the unexplained random portion of the model.

For continuous measurements such as heart rate variability (HRV), heart rate (HR), and skin conductance level (SCL), an effects model was created to analyze the restorative effects of the green wall, outdoor view, and the combination of green wall and outdoor view environments on these physiological indicators during recovery (Model 2).

$\begin{array}{l} \left(\log\right)\;Yij=\beta 0+\beta 1^{*}\;\mathrm{environment}+\beta 2^{*}\;\mathrm{time}\\ +\beta 3^{*}\;{\mathrm{environment}}^{*}\;\mathrm{time}+eij+\mu i \end{array}$ (2)

In this model:

(log) Yij: The dependent variable in the model, representing the physiological parameter (e.g., HR, HRV, or SCL) for participant i at time point j.

β0: Intercept term in the model, representing the average (log) Yij value at time 0 when the environment is the reference environment.

β1: The coefficient for environmental factors, representing the impact of the environment on (log) Yij. It measures the average differences in (log) Yij under different environmental conditions.

β2: The coefficient for time factors, representing the impact of time on (log) Yij. It measures the trend of (log) Yij over time.

β3: The coefficient for the interaction between environment and time, representing the impact of the interaction between environment and time on (log) Yij. It assesses whether (log) Yij changes with time differently in different environments.

eij: The error term in the model, representing the unexplained random variation.

μi: The random effect in the model, representing the random variation in the intercept for each participant i. It accounts for individual differences between participants.

This model illustrates the impact of environment and time on physiological data while considering individual differences and random errors. The model was fitted using statistical software (SPSS), and its significance was examined to investigate the effects of environment and time on HRV, HR, and SCL data.

To test the factors related to the recovery of participants’ physiological data back to baseline in different environmental conditions, a Cox proportional hazards model was established (Model 3), studying the time-event relationship:

$\begin{array}{l} \mathrm{Surv}\left(\mathrm{time}:to:\mathrm{recovery},\mathrm{complete}:\mathrm{recovery}\right)\\ \;\sim \mathrm{environment} \end{array}$ (3)

Where:

time_to_recovery: The time it took for participants to recover to their baseline state.

complete_recovery: An event indicator variable, representing complete recovery (1 indicating complete recovery, 0 indicating incomplete recovery).

Environment: Represents only environmental factors used to assess their impact on complete recovery under different environmental conditions.

In this model, the time it took for each participant to restore their physiological indicators to baseline was recorded. Complete recovery was defined as the participant’s physiological indicators returning to baseline levels. Data for participants who did not achieve complete recovery within the 5-min recovery period were excluded. Participants who did not exhibit physiological stress responses during the experiment were also excluded. The Cox model estimated the hazard ratio for complete recovery and its confidence interval, measuring the relative likelihood of participants in the biophilic environment compared to the non-biophilic environment achieving complete recovery during the recovery period. The hazard ratio (HR) represents the ratio at which participants in the biophilic environment achieve complete recovery before participants in the non-biophilic environment and calculates the probability of first recovery (P) = HR / (1 + HR). All analyses were conducted using Kaplan–Meier and Cox regression models in SPSS.

## 3 Results

The results are presented in five sections. First, demographic information and physiological data measurements are described. To test the effectiveness of random assignment, a variance analysis was conducted. To determine the effectiveness of stress induction tasks, independent sample t-tests were used for normally distributed data, and the Wilcoxon signed-rank test was employed for non-normally distributed data. Second, the impact of green walls, outdoor view, and the combination of green walls and outdoor view on instantaneous blood pressure (BP) and anxiety states (STAI) is examined. Third, the effects of green walls, outdoor view, and the combination on changes in continuous physiological measurements, including heart rate variability (HRV), heart rate (HR), and skin conductance level (SCL) before and after exposure, are explored. Fourth, the relationship between continuous physiological measurements and recovery time is investigated. Finally, self-reported and perceived restorativeness changes are discussed (50).

### 3.1 Demographics, baseline measures and stressor

Table 1 presents the characteristics of the 23 participants and 92 visits. The average age of the participants was 62.7 ± 7.4 years, with 69.6% being female and 30.4% male. About 70.6% of the participants reported their health status as excellent or very good. 90.2% of the participants reported having good sleep, while 16.3% had consumed caffeinated beverages before the experiment. The majority of participants reported feeling no stress, with 62% indicating that they either really liked or somewhat liked natural environments. Only 4.3% of participants had a history of smoking, and 1% had a history of alcohol consumption. None of the participants engaged in high-intensity exercise. Participants rated their preferences for non-biophilic environments, green walls, outdoor view, and their combinations as 1.9 ± 1.0, 2.0 ± 1.1, 2.9 ± 0.9, and 3.2 ± 1.0, respectively, on a total score of 5. The perceived connection with nature for these environments was rated as 5.9 ± 3.7 for non-biophilic, 6.4 ± 2.8 for green walls, 7.4 ± 2.0 for outdoor view, and 8.8 ± 1.7 for the combination. Participants perceived a stronger connection to nature in the combination environment, followed by outdoor view, green walls, and the lowest connection was perceived for non-biophilic environments (Table 2).

**Table 1:** Indicator.

Indicator	
BP	Blood Pressure
SBP	Systolic Blood Pressure
DBP	Diastolic Blood Pressure
HR	Heart Rate
HRV	Heart Rate Variability
VR	Virtual Reality
RMSSD	Root Mean Square of Successive Differences
SCL	Skin Conductance Level
LF/HF	Low-Frequency to High-Frequency Ratio (used in heart rate variability analysis)
STAI	State–Trait Anxiety Inventory
ART	Attention Restoration Theory
SRT	Stress Reduction Theory

Abbreviations and full names of various indicators.

**Table 2:** Characteristics of study participants (*n* = 23) at baseline of the experiment.

Baseline	Count	Mean + SD or n(%)			
Environment		Non-Biophilic	Green Wall	Outdoor view	Combination
Participants	23	23	23	23	23
Age	61.9 ± 1.5	61.9 ± 1.5	61.9 ± 1.5	61.9 ± 1.5	61.9 ± 1.5
Gender					
Male	28(30.4)	7(30.4)	7(30.4)	7(30.4)	7(30.4)
Female	64(69.6)	16(69.6)	16(69.6)	16(69.6)	16(69.6)
Self-Reported Health					
Excellent	6(6.5)	3(13.0)	1(4.3)	1(4.3)	1(4.3)
Very Good	59(64.1)	13(56.5)	15(65.2)	15(65.2)	16(69.6)
Good	24(26.1)	6(26.1)	6(26.1)	7(30.4)	5(21.7)
Fair	2(2.2)	1(4.3)	1(4.3)	0(0.0)	1(4.3)
Poor	0(0.0)	0(0.0)	0(0.0)	0(0.0)	0(0.0)
Sleep					
Good	83(90.2)	21(91.3)	20(87.0)	21(91.3)	21(91.3)
Not Good	9(9.8)	2(8.7)	3(13.0)	2(8.7)	2(8.7)
Caffeine Intake					
Yes	15(16.3)	4(17.4)	5(21.7)	2(8.7)	4(17.4)
No	77(83.7)	19(82.6)	18(78.3)	21(91.3)	19(82.6)
Self-Reported intensity Level	1.2 ± 0.5	1.1 ± 0.5	1.2 ± 0.6	1.1 ± 0.5	1.2 ± 0.5
Preference for Nature					
Extremely Dislike	1(1.1)	1(4.3)	0(0.0)	0(0.0)	0(0.0)
Slightly Dislike	7(7.6)	1(4.3)	2(8.7)	2(8.7)	2(8.7)
Neutral	27(29.3)	6(26.1)	7(30.4)	7(30.4)	7(30.4)
Quite Like	49(53.3)	13(56.5)	12(52.2)	12(52.2)	12(52.2)
Extremely Like	8(8.7)	2(8.7)	2(8.7)	2(8.7)	2(8.7)
Smoking					
Yes	4(4.3)	1(4.3)	1(4.3)	0(0.0)	1(4.3)
No	88(95.7)	22(95.7)	22(95.7)	23(100.0)	22(95.7)
Alcohol					
Yes	1(1.0)	0(0.0)	0(0.0)	1(4.3)	0(0.0)
No	91(99.0)	21(91.3)	23(100.0)	22(95.7)	23(100.0)
High-Intensity Exercise					
Yes	0(0.0)	0(0.0)	0(0.0)	0(0.0)	0(0.0)
No	92(100.0)	23(100.0)	23(100.0)	23(100.0)	23(100.0)
Connection to Nature	7.1 ± (2.8)	5.9 ± 3.7	6.4 ± 2.8	7.4 ± 2.0	8.8 ± 1.7
Degree of Liking	2.5 ± (1.1)	1.9 ± 1.0	2.0 ± 1.1	2.9 ± 0.9	3.2 ± 1.0

Percentage (numeric value). Self-reported intensity of exercise in the past 4 weeks, from 1 “Not at all intense” to 5 “Very intense.”

**Figure 3:**
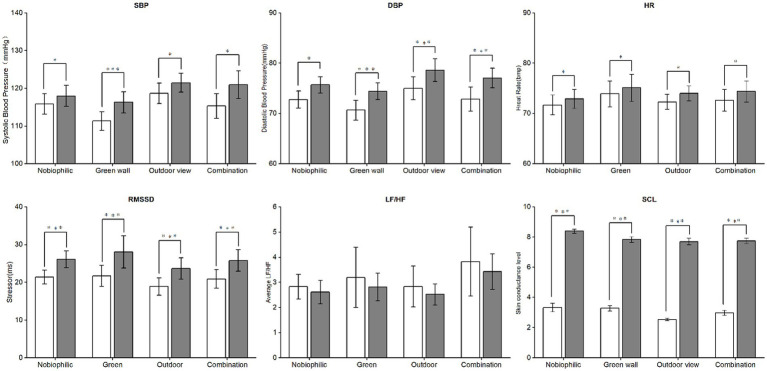
Comparing the significance of the average value change in physiological data before and after stress, *p* < 0.05 indicates a significant difference, while *p* < 0.001 indicates an extremely significant difference.

In addition, the baseline physiological indicators were similar across the four conditions with no significant differences (Table 3). Figure 3 and Table 3 present the mean and median values of participants’ physiological measurements at baseline, post-stressor, and during recovery. Results from paired-sample t-tests and Wilcoxon signed-rank tests indicate that participants’ physiological stress levels significantly increased after experiencing the stressor. Furthermore, the analysis of variance (ANOVA) showed no significant differences in blood pressure, STAI scores, SCL, HR, and HRV among the four groups. Therefore, there were no significant differences in stress and anxiety levels among the four groups.

**Table 3:** ANOVA analysis of baseline physiological indicators.

Indicator		Nonbiophilic	Green wall	Outdoor view	Combination
SBP	F	1.06	0.9	1.13	0.7
	P	0.35	0.41	0.33	0.5
DBP	F	1.53	2.13	1.29	1.8
	P	0.22	0.15	0.26	0.19
HR	F	0.21	0.11	0.70	0.41
	P	0.65	0.74	0.41	0.53
LF/HF	F	0.10	0.08	0.11	0.68
	P	0.75	0.78	0.74	0.80
RMSSD	F	2.82	1.54	1.68	1.72
	P	0.1	0.22	0.21	0.2
SCL	F	1.64	0.55	0.21	0.01
	P	0.22	0.47	0.65	0.96

When F is larger and *p* < 0.05, it indicates a significant difference. The F-value is a statistical measure in analysis of variance (ANOVA) used to assess whether the means among different groups are significantly different.

### 3.2 Effect of biophilic environments on pre-post changes of BP, STAI

**Figure 4:**
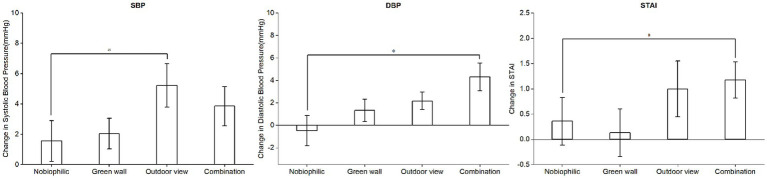
The changes in SBP, DBP, and STAI scores from the period of stress induction to the post-recovery phase. *p* < 0.05 indicates a significant difference.

In contrast to non-biophilic settings, participants exhibited a more substantial reduction in systolic blood pressure (SBP) and diastolic blood pressure (DBP) within biophilic environments. The decline in systolic blood pressure (SBP) surpassed that of diastolic blood pressure (DBP). Furthermore, systolic blood pressure (SBP) displayed a significant correlation with exposure to outdoor views, while diastolic blood pressure (DBP) showed a significant association with the combined biophilic environments. In detail, participants exhibited a decrease in systolic blood pressure (SBP) of 1.34 mmHg in the non-biophilic environment, which was less than the reductions observed in the biophilic environments. Specifically, within the green wall environment, participants experienced a reduction in SBP of 2.04 mmHg, while in the outdoor view environment, SBP decreased significantly by 5.22 mmHg. In the combined biophilic environment, SBP decreased by 3.86 mmHg, with a notable reduction observed particularly in the outdoor view environment (Figure 4). Conversely, participants’ diastolic blood pressure (DBP) increased by 0.48 mmHg in the non-biophilic environment but showed a decrease in the biophilic environments. More precisely, within the green wall, outdoor view, and combination environments, DBP decreased by 1.33 mmHg, 2.17 mmHg, and 4.32 mmHg, respectively. Notably, the most substantial reduction in DBP was observed in the outdoor view and combination environments (Figure 4).

Participants exhibited lower STAI scores post-recovery in all four conditions, signifying anxiety alleviation following stress induction. The most notable reduction in STAI scores occurred within the combined environment (Figure 4). When contrasting biophilic and non-biophilic settings (95% CI: −1.92, −1.58), a more substantial decline in STAI scores was observed in biophilic environments. Specifically, the outdoor view (95% CI: −1.61, −0.61) and combined (95% CI: −2.17, −1) conditions demonstrated marked decreases, with respective reductions of 1 and 1.17, while the green wall environment displayed a comparatively modest decline (95% CI, −1.96, −1.83).

### 3.3 Effect of biophilic environments on recovery rates of HRV, HR and SCL

**Figure 5:**
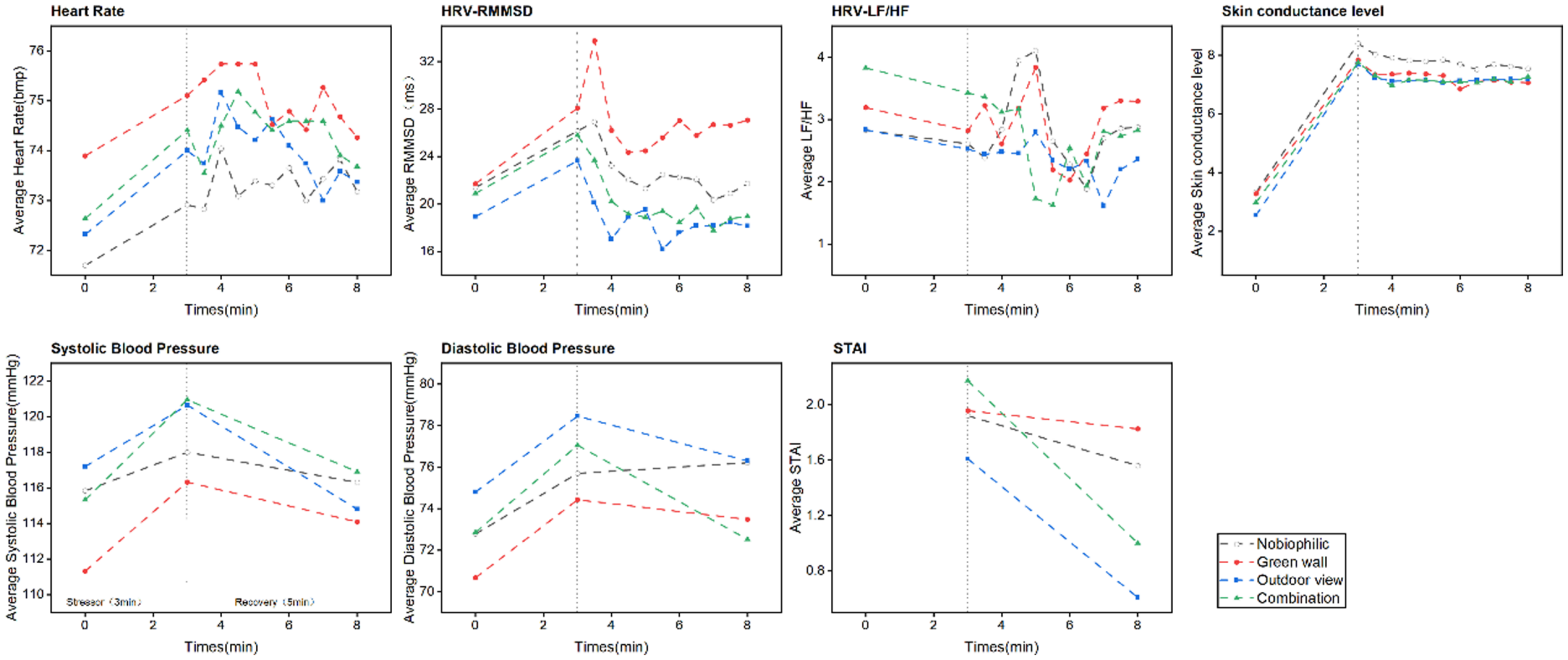
Mean variations in heart rate (HR), heart rate variability measured by RMSSD, heart rate variability in the low-frequency to high-frequency ratio (LF/HF), skin conductance level (SCL), systolic blood pressure (SBP), diastolic blood pressure (DBP), and State–Trait Anxiety Inventory (STAI) scores during the stress and recovery periods.

**Figure 6:**
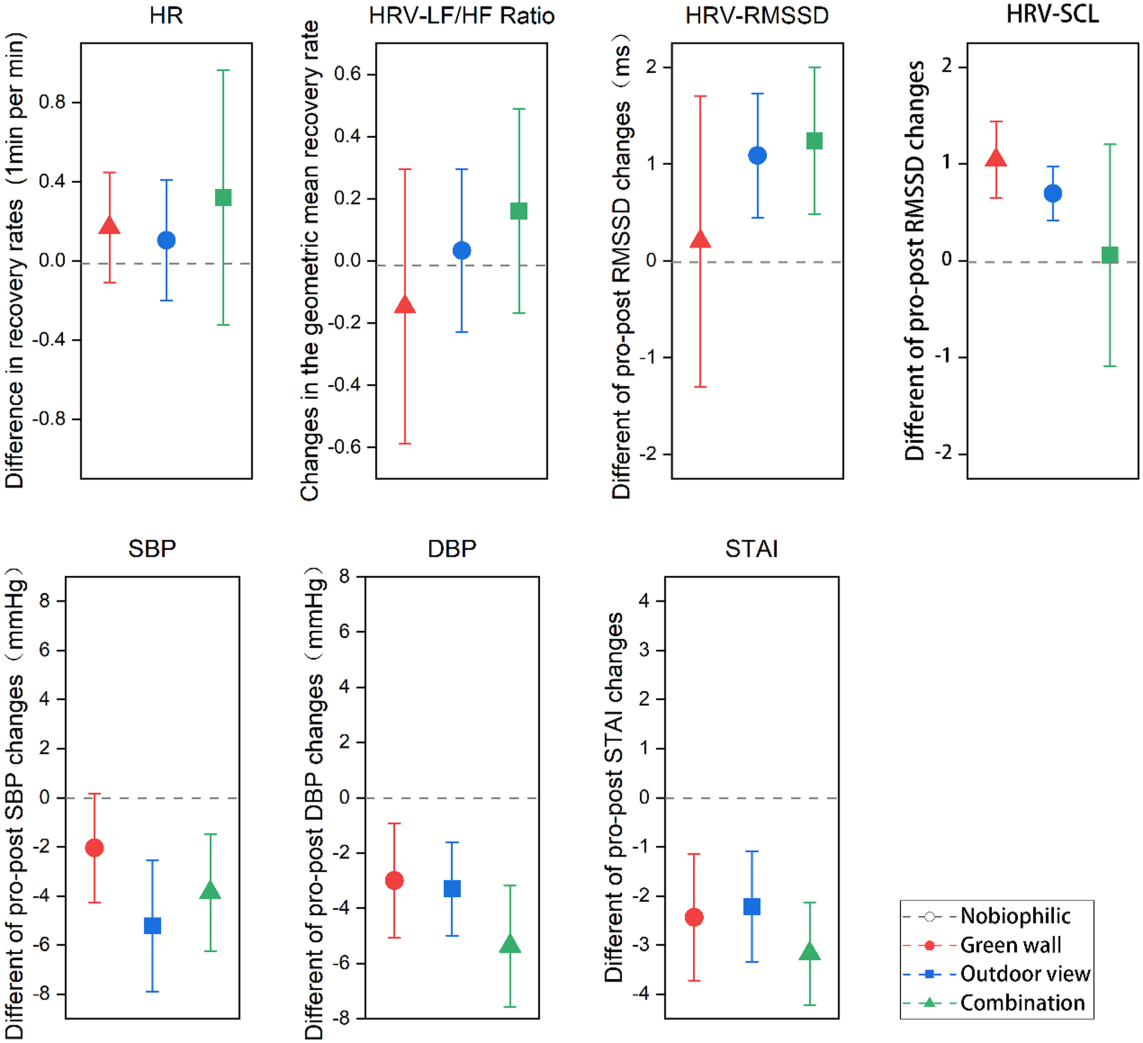
Differences in recovery rates of heart rate (HR), percentage changes in the geometric mean recovery rate of heart rate variability (HRV) and skin conductance level (SCL), and differences in pre-post changes in blood pressure (SBP & DBP) and state–trait anxiety inventory (STAI) score in biophilic environments versus those in non-biophilic during the 6-min recovery period. RMSSD, Root mean square of the successive differences; LF/HF, Ratio of low frequency to high frequency; Error bars depict 95% confidence interval.

Figure 5 depicts the mean alterations in participants’ physiological indicators throughout the 8-min experimental procedure. Figure 6 illustrates alterations in the average recovery rates of Heart Rate Variability (HRV) as measured by Root Mean Square of Successive Differences (RMSSD) and Skin Conductance Level (SCL) during the 5-min recovery period in both biophilic environments (green wall, outdoor view, combination) and non-biophilic environments. The study revealed that participants exhibited a higher RMSSD recovery rate in biophilic environments. Specifically, in the biophilic outdoor view environment, RMSSD demonstrated a significant recovery effect. In the early phase of recovery, RMSSD exhibited a recovery rate of 9% (95% CI: −4.3 to 22.4%), while in the mid-phase, it registered a rate of 2.1% (95% CI: −2.6 to 6.9%), with no significant recovery effect observed in the late phase. Furthermore, the impact of RMSSD recovery varied across the three phases. In the early phase, the geometric mean growth rate of RMSSD in the green wall environment was 4.3% (95% CI: −5.6 to 14.17%), whereas in the outdoor view condition, it was faster at 4.7%, and in the combination condition, it reached 9% (95% CI: −4.3 to 22.4%). During the mid-phase, the green wall environment demonstrated a growth rate of 5.6% (95% CI: 0.56 to 11.4%), outpacing the outdoor view rate of 2.1% (95% CI: −2.6 to 6.9%) and the combination environment rate of 2.2% (95% CI: −0.8 to 5.2%). In the late phase, recovery rates of RMSSD decreased in all three environments, indicating no observable recovery effect. However, there were no statistically significant differences in the recovery rates of LF/HF ratio, Heart Rate (HR), and SCL between the biophilic and non-biophilic environments.

### 3.4 Effect of biophilic environments on time to complete recovery

**Figure 7:**
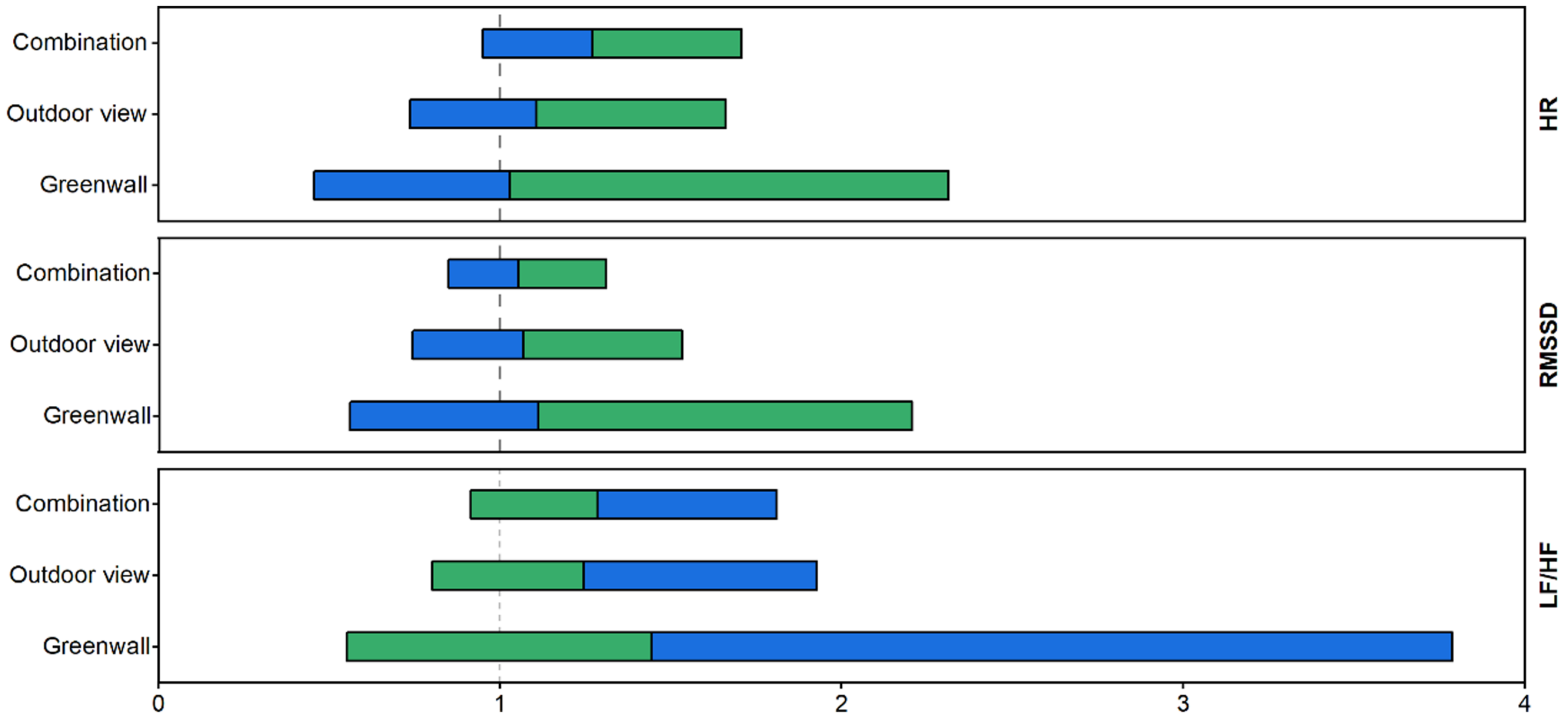
The hazard ratio (HR) represents the ratio at which participants in the biophilic environment achieve complete recovery before participants in the non-biophilic environment and calculates the probability of first recovery (P) = HR / (1 + HR). All analyses were conducted using Kaplan–Meier and Cox regression models in SPSS.

Figure 7 presents the estimated hazard ratios concerning the full recovery of physiological parameters in biophilic environments compared to their non-biophilic counterparts during the 5-min recovery phase. Participants whose Skin Conductance Level (SCL) measurements did not return to baseline during this recovery period were excluded from the analysis. Consequently, the Cox model analysis was conducted with a sample size of *n* = 59 for Heart Rate (HR), *n* = 61 for Root Mean Square of Successive Differences (RMSSD), and *n* = 38 for Low-Frequency to High-Frequency Heart Rate Variability ratio (LF/HF ratio). The focus of this analysis was on the estimated hazard ratios for complete recovery in biophilic environments. In biophilic environments, the estimated hazard ratios for complete RMSSD recovery were all greater than 1. Specifically, the values were 1.11 (95% CI: 0.56 to 2.21) for the green wall, 1.07 (95% CI: 0.74 to 1.53) for the outdoor view, and 1.06 (95% CI: 0.85 to 1.31) for the combination condition. These values imply that participants had a 53, 52, and 51% likelihood of achieving complete RMSSD recovery in the green wall, outdoor view, and combination conditions, respectively. Likewise, in biophilic environments, the estimated hazard ratios for complete HR recovery were all greater than 1, with values of 1.03 (95% CI: 0.46 to 2.31) for the green wall, 1.12 (95% CI: 0.74 to 1.67) for the outdoor view, and 1.27 (95% CI: 0.95 to 1.71) for the combination condition. This suggests that participants had a 51, 53, and 56% chance of experiencing complete HR recovery in the green wall, outdoor view, and combination conditions, respectively. Lastly, in biophilic environments, the estimated hazard ratios for complete LF/HF ratio recovery were all greater than 1, with values of 1.44 (95% CI: 0.55 to 3.78) for the green wall, 1.24 (95% CI: 0.80 to 1.93) for the outdoor view, and 1.29 (95% CI: 0.92 to 1.81) for the combination condition. These values indicate that participants had a 59, 55, and 56% chance of achieving complete LF/HF ratio recovery in the green wall, outdoor view, and combination conditions, respectively. Additionally, in the combination and outdoor environments, the estimated hazard ratios for complete recovery exhibited more stability, suggesting that participants’ complete recovery times were more consistent in these conditions.

**Figure 8:**
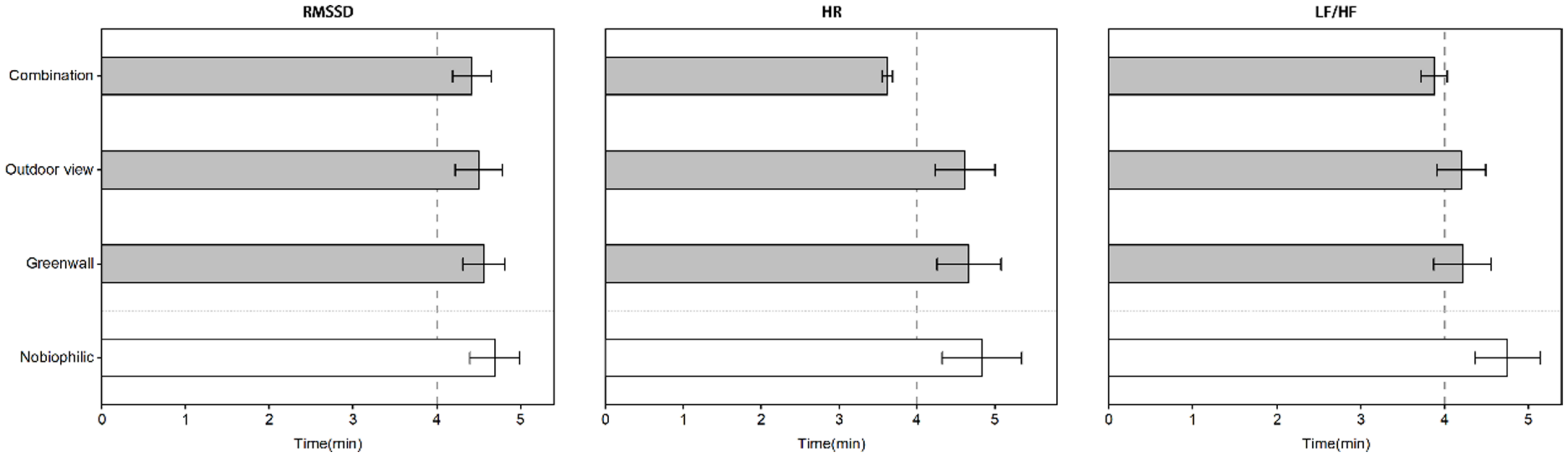
The duration required for physiological parameters to fully recover in biophilic environments compared to non-biophilic environments during the 5-min post-stress recovery period.

Figure 8 illustrates the duration required for complete recovery of physiological parameters in biophilic environments as opposed to non-biophilic environments during the 5-min recovery period. Among the physiological parameters analyzed, including Heart Rate (HR), Root Mean Square of Successive Differences (RMSSD), and Low-Frequency to High-Frequency Heart Rate Variability ratios (LF/HF ratios), the combination biophilic environment exhibited the shortest recovery times. The investigation revealed that within biophilic environments, participants experienced significantly shorter recovery times for LF/HF ratios. In the combination biophilic environment, the recovery time for HR was 3.60 (95% CI: 0.07 to 0.21), which was markedly shorter when contrasted with the non-biophilic environment’s HR recovery time of 4.95 (95% CI: 0.61 to 1.94). This underscores that individuals in the combination biophilic environment achieved a more rapid HR recovery in comparison to those in the non-biophilic environment.

### 3.5 Self-reported, perceived restorativeness, connection to nature

**Figure 9:**
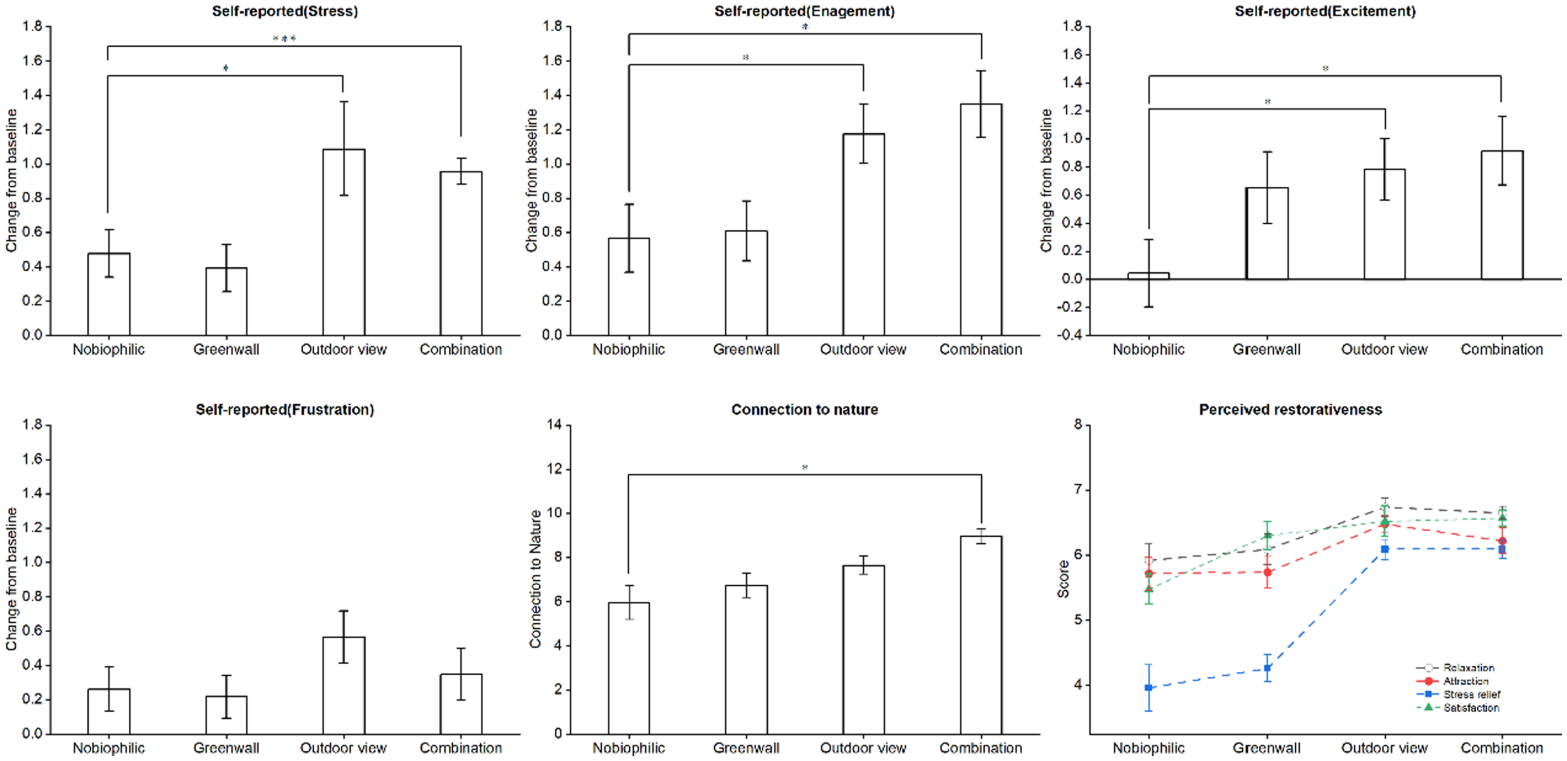
The alterations and statistical significance observed in self-reported measures, perceived restorativeness, and the connection to nature both before and after exposure to stress.

The results indicate that, compared to non-biophilic environments, participants experienced higher levels of excitement in biophilic environments (Figure 9). Specifically, the effect was most significant in the combination biophilic environment, followed by the outdoor view, and to a lesser extent, the green wall environment. Participants’ self-reported emotional well-being also improved in the green wall environment. In biophilic environments, participants experienced greater reductions in stress and frustration levels and reported higher levels of engagement and excitement. These findings align with the physiological results and suggest that emotional well-being improves in biophilic environments (green wall, outdoor view, and combination).

The results from the Perceived restorativeness scale show that biophilic environments were more appealing to participants and scored higher in terms of perceived stress relief, relaxation, and satisfaction (Figure 9). According to the table, the outdoor view environment demonstrated the most significant restorative effects. Participants reported feeling more relaxed (6.74, 95% CI: 6.44, 7.04), more attracted (6.48, 95% CI: 6.19, 6.77), experienced greater stress relief (6.09, 95% CI: 5.77, 6.4), and higher satisfaction (6.52, 95% CI: 6.04, 7.01) in the outdoor view environment. Participants also reported a reduction in negative emotions and an increase in positive emotions. These findings suggest that biophilic environments, especially outdoor view, have a positive impact on participants’ emotional well-being and sense of restoration.

Participants generally perceive a closer connection to nature in biophilic environments (Figure 9). Specifically, participants rated their connection to nature as 5.96 (95% CI: 4.38–7.53) in non-biophilic environments, 6.74 (95% CI: 5.61–7.87) in green wall environments, 7.65 (95% CI: 6.81–8.49) in outdoor view environments, and 8.96 (95% CI: 8.29–9.63) in combination environments. Notably, the sense of connection was most pronounced in combination environments.

## 4 Discussion

**Figure 10:**
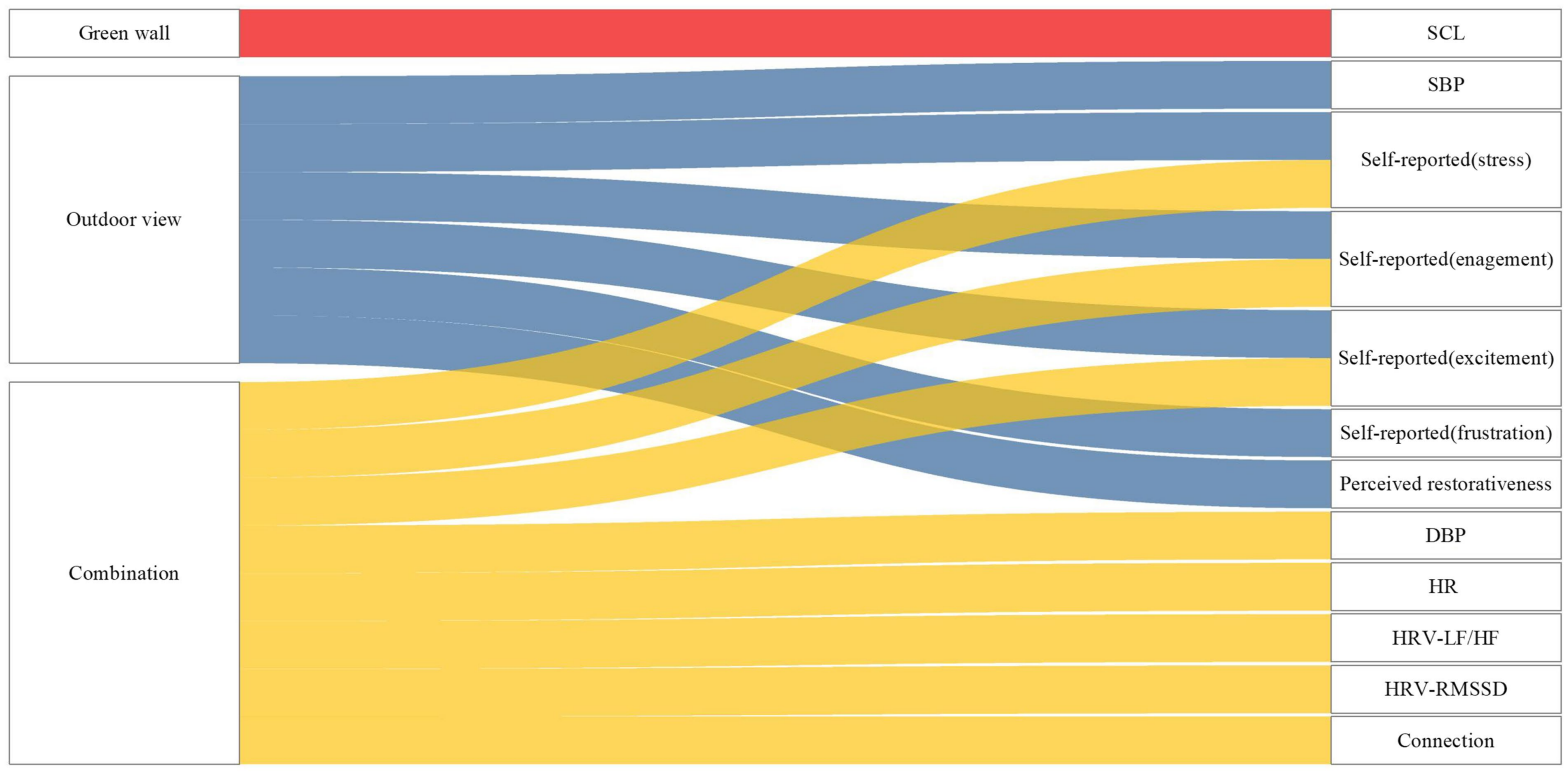
The relationship between significant physiological indicators and the environment.

In this study, a total of 23 participants took part in 92 sessions. Participants were randomly assigned to one of four virtual indoor environments over the course of 4 weeks, at the same time each week. These environments included a non-biophilic baseline environment and three biophilic environments: a green wall, an outdoor view, and a combination environment. The research findings demonstrate that, compared to the non-biophilic environment, the green wall, outdoor view, and the combination of both, exhibited higher levels of stress reduction and anxiety recovery, indicating superior stress recovery in biophilic environments (Figure 10). Different nature connectedness indoor environments had varying impacts on both physiological and psychological responses. Notably, the outdoor view was particularly effective in reducing anxiety, while physiological indicators exhibited distinct changes in different nature connectedness environments. Specifically, systolic blood pressure (SBP) showed the most significant reduction in the combination environment, while LF/HF, RMSSD, HR, and diastolic blood pressure (DBP) were most influenced by the outdoor environment. Participants experienced shorter recovery times in the combination environment. However, in the green wall environment, while there was some stress reduction in both physiological and psychological anxiety levels during the recovery period, the changes were not statistically significant. During the recovery period, the study found that in the five-minute recovery window, the most substantial effects occurred during the first 3.5 min, with minimal recovery effects during the last 1.5 min.

### 4.1 Physiological responses to virtual settings

The physiological data results obtained from linear models, mixed-effects models, and Cox models indicate that participants experienced better stress recovery effects in nature connectedness indoor environments. When subjects were exposed to natural surroundings, their recovery was faster and more complete (10). Participants exhibited higher rates of recovery in RMSSD, LF/HF, HR, SCL, SBP, and DBP in biophilic indoor environments. Viewing natural landscapes had a positive physiological recovery effect (51), with green walls being effective in mitigating indoor stress and their association with a decrease in participants’ blood pressure (52, 53). Overall differences in participants’ physiological responses to experiencing indoor environments with nature elements versus non-biophilic environments were observed, with a reduction in systolic and diastolic blood pressure in the nature connectedness indoor environment compared to the non-biophilic environment. The absence of natural landscapes indoors may have a negative impact on individuals, which could be addressed by providing outdoor views through windows (54). The study found that, except for SCL, the average values of other physiological indicators, including RMSSD, LF/HF, HR, SBP, and DBP, recovered to baseline or below baseline levels, indicating full recovery by participants. Additionally, hazard ratios derived from Cox risk models suggest that participants’ physiology had fully recovered. Furthermore, participants in biophilic environments exhibited faster recovery rates in LF/HF, RMSSD, and HR, requiring less time to recover, demonstrating the restorative potential of nature connectedness indoor environments.

### 4.2 Psychological responses to virtual settings

This section discusses the positive effects of nature connectedness environments on participants’ anxiety relief. The results indicate significant changes in participants’ STAI (State–Trait Anxiety Inventory), self-reported emotional tests, and recovery tests in nature connectedness environments compared to non-biophilic environments. The study findings suggest the influence of nature interventions on individual perceptual outcomes, such as preferences, perceived restorativeness, and satisfaction, consistently plays a role (55). After a 5-min recovery in a nature connectedness environment, participants experienced a decrease in STAI scores, improved self-reported emotional states, and increased Perceived restorativeness. This indicates that nature connectedness environments can effectively promote participants’ recovery.

### 4.3 Differential effects in three biophilic environments

The study revealed that three different nature connectedness indoor environments had varying restorative effects on physiological stress indicators and anxiety levels. Watching outdoor view was found to be most effective in alleviating physiological stress compared to the other conditions. Previous research has indicated that the recovery of parasympathetic nervous system activity is significantly higher after viewing outdoor natural landscapes, and visual contact with outdoor natural scenery can improve the recovery process following stressors (56). Positive effects on recovery of attention, stress reduction, overall health, and well-being are associated with visual connections to nature (57). The outdoor view had a notably direct impact on stress reduction (58). In combination with environmental factors, it had a greater impact on anxiety reduction (as measured by self-reported emotional tests, restoration scales, and self-assessment of connection with nature). Following the outdoor view, green walls, and outdoor view, both provided participants with visual connections to nature, but the content of the outdoor view was richer, including features like natural light through windows and the presence of large trees outside. The study results suggest that, for older adults individuals, outdoor view environments are more conducive to physiological recovery compared to green wall environments. In combination with the environment, outdoor view are more effective in reducing anxiety levels. The green wall environment, while still contributing to stress reduction and decreased anxiety levels among older adults individuals, did not yield significant results, and there was substantial variation in the preference for green walls among different older adults individuals. Since the experiment only presents a square frame containing some plants, without displaying natural forms and shapes on these walls. Compared to rigid geometric forms, people have a more positive response to natural forms and geometric structures (such as fluidity of fractal growth and geometry) (59, 60). Therefore, future research will consider incorporating more natural forms into the design to further enhance the ecological benefits and human responses of green walls.

## 5 Conclusion

This study evaluates the impact of green walls, outdoor views, and their combination on stress and anxiety levels in older adults by quantifying their psychological and physiological responses. Employing virtual reality technology and wearable physiological sensors, the analysis encompasses metrics such as blood pressure (BP), heart rate (HR), heart rate variability (HRV), skin conductance level (SCL), State–Trait Anxiety Inventory (STAI), Recovery Scale, and self-reported emotional assessments to assess participants’ stress and anxiety levels.

The findings indicate that, relative to non-biophilic environments, environments incorporating green walls, outdoor views, or their combination exert a more pronounced influence on stress reduction and anxiety alleviation in older adults. Specifically, systolic blood pressure is notably associated with outdoor view environments, while diastolic blood pressure significantly correlates with combined environments. HR, RMSSD, and LF/HF exhibit significant variations in combined environments, substantial variations in outdoor view environments, and minimal variations in green wall environments. The likelihood of complete recovery in HR, RMSSD, and LF/HF is highest in green wall environments, although not significantly different from outdoor view or combined environments. Alterations in STAI scores and self-reported changes among older adults are highly significant in combined environments and significantly divergent in outdoor view environments.

In summary, outdoor views and combined environments effectively alleviate stress and significantly ameliorate anxiety, while green walls also mitigate stress and anxiety to a lesser extent. Consequently, when selecting and designing indoor communal environments in older adults care facilities, it is advisable to prioritize the incorporation of high-quality outdoor views.

## Data availability statement

The raw data supporting the conclusions of this article will be made available by the authors, without undue reservation.

## Ethics statement

The studies involving humans were approved by Southwest Jiaotong University ethics committee. The studies were conducted in accordance with the local legislation and institutional requirements. Written informed consent for participation in this study was provided by the participants’ legal guardians/next of kin.

## Author contributions

SX: Conceptualization, Data curation, Formal analysis, Investigation, Methodology, Resources, Software, Visualization, Writing – original draft, Writing – review & editing. XH: Funding acquisition, Supervision, Validation, Writing – review & editing, Project administration.
